# EVI1 oncoprotein expression and CtBP1-association oscillate through the cell cycle

**DOI:** 10.1007/s11033-020-05829-1

**Published:** 2020-09-26

**Authors:** Roberto Paredes, Marion Schneider, Stella Pearson, Hsiang Yin Teng, James R. Kelly, Andrew Pierce, Tim C. P. Somervaille, Anthony D. Whetton, Stefan Meyer

**Affiliations:** 1 Stem Cell and Leukaemia Proteomics Laboratory, Division of Cancer Sciences, Faculty of Biology, Medicine and Health, University of Manchester, Manchester, UK; 2 Manchester Academic Health Science Centre, National Institute for Health Research Biomedical Research Centre, Manchester, UK; 3 Leukaemia Biology Group, CRUK Manchester Institute, Manchester, UK; 4 Stoller Biomarker Discovery Centre, University of Manchester, Manchester, UK; 5 Department of Paediatric Haematology and Oncology, Royal Manchester Children’s Hospital, Manchester, UK; 6 c/o Academic Unit of Paediatric Oncology, Young Oncology Unit, The Christie NHS Foundation Trust, Christie Hospital, Wilmslow Road, Manchester, M20 6XB UK

**Keywords:** EVI1, CtBP1, Cell cycle, AML

## Abstract

Aberrantly high expression of *EVI1* in acute myeloid leukaemia (AML) is associated with poor prognosis. For targeted treatment of *EVI1* overexpressing AML a more detailed understanding of aspects of spatiotemporal interaction dynamics of the EVI1 protein is important. EVI1 overexpressing SB1690CB AML cells were used for quantification and protein interaction studies of EVI1 and ΔEVI1. Cells were cell cycle-synchronised by mimosine and nocodazole treatment and expression of EVI1 and related proteins assessed by western blot, immunoprecipitation and immunofluorescence. EVI1 protein levels oscillate through the cell cycle, and EVI1 is degraded partly by the proteasome complex. Both EVI1 and ΔEVI1 interact with the co-repressor CtBP1 but dissociate from CtBP1 complexes during mitosis. Furthermore, a large fraction of EVI1, but not ΔEVI1 or CtBP1, resides in the nuclear matrix. In conclusion, EVI1- protein levels and EVI1-CtBP1 interaction dynamics vary though the cell cycle and differ between EVI1 and ΔEVI1. These data ad to the functional characterisation of the EVI1 protein in AML and will be important for the development of targeted therapeutic approaches for EVI1-driven AML.

**Electronic supplementary material:**

The online version of this article (10.1007/s11033-020-05829-1) contains supplementary material, which is available to authorized users.

## Introduction

**Fig. 1:**
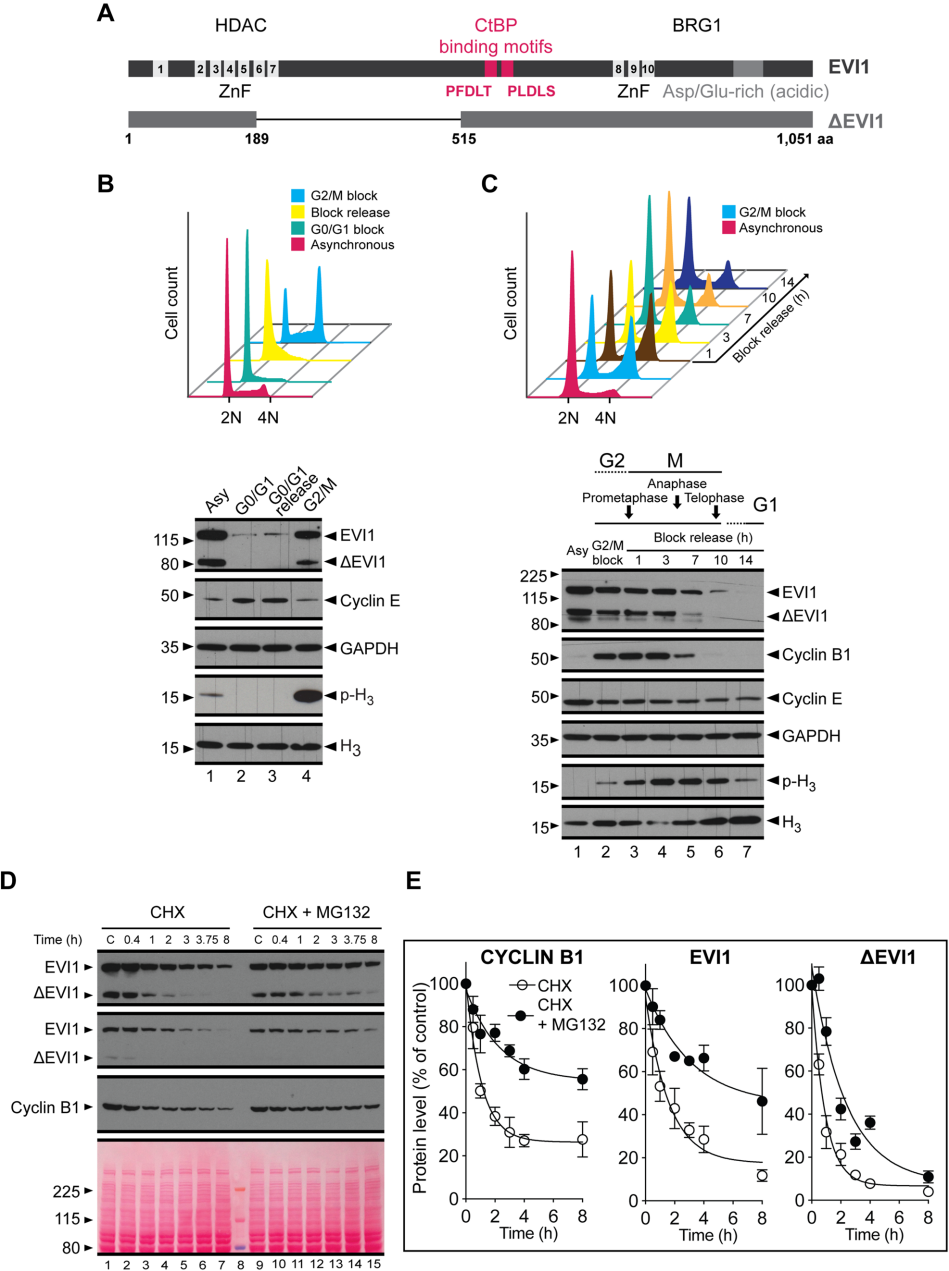
EVI1 degradation during mitosis. **a** Schematic illustration of the EVI1 and ΔEVI1 isoforms with numbered zinc finger motifs (ZnF), CtBP1 binding motifs (red) and presumed regions of interaction with HDAC and BRG1 proteins. **b** Upper panel: propidium iodine (PI) cell cycle FACS profile of SB1690CB AML cells: asynchronous cells (magenta), synchronised in G0/G1 with Mimosine treatment for 18 h (turquoise) and then block-released for 7 h (yellow); synchronised in G2/M with a nocodazole treatment for 24 h (cyan). Lower panel: Western blot analysis of EVI1 isoforms in whole cell lysates from cells arrested as in the top panel. GAPDH and H_3_ were used as loading controls, CYCLIN E2 as cell cycle phase marker and Ser10-phospho H_3_ as mitotic marker. **c** Upper panel: PI cell cycle FACS profile of SB1690CB AML cells: Asynchronous cells (magenta), synchronised in G2/M with a Nocodazole treatment for 24 h (cyan) and then block-released for 1 h (brown), 3 h (yellow), 7 h (turquoise), 10 h (orange) and 14 h (blue). Lower panel: Western blot analysis as in B, with the addition of CYCLIN B1 as cell cycle marker control. **d** Western blot analysis of EVI1 isoforms in SB1690CB AML cells treated with 10 µg/ml cycloheximide (CHX) alone or in combination with 5 µM MG-132 for duration as indicated. Due to differences in expression levels of EVI1 isoforms, two exposures are shown and used for quantification purposes. CYCLIN B1 was used as a control and Ponceau S stain shown for protein loading. **e** Quantitation of EVI1 isoforms and CYCLIN B1 protein levels from three independent experiments. (Color figure online)

Aberrantly high expression of *EVI1* in acute myeloid leukaemia (AML) is commonly caused by chromosomal aberrations involving the *MECOM* (MDS-EVI1 complex) locus at 3q26 and associated with poor outcome [1, 2]. In AML, the overexpressed 1051 amino acid (aa) EVI1 protein can be co-expressed with the shorter ΔEVI1 isoform, which lacks a 324 aa sequence region (aa190-514), including the 6th and 7th zinc finger of the N-terminal zinc finger domain (Fig. 1a). The MDS-EVI1 isoform is usually not expressed at elevated levels [2]. DNA binding sites of the ΔEVI1 isoform largely overlap with those of EVI1, but it lacks in vivo transforming ability characteristic for EVI1 [3, 4]. While the reliance of EVI1 on interaction with other transcriptionally active proteins, e.g. CtBP1 [5], has been recognised and provides potential angles for therapeutic approaches, spatiotemporal dynamics of the EVI1 protein isoforms in AML are incompletely understood, but would be important for the development of EVI1-targeted therapeutic approaches. Here, we report on data that uncover cell cycle and isoform specific localisation and interaction dynamics of EVI1 and ΔEVI1.

## Materials and methods

### Cell culture

The EVI1-overexpressing AML cell line SB1690CB was maintained as described previously [6]. Further details of cell lines in supplementary material. For cell cycle arrest in G0/G1 cells were treated with mimosine (200 µM, Sigma) for 18 h. For G2/M arrest nocodazole 200 ng/mL was used (Sigma) for 24 h. Cells were released from cell cycle arrest by washing and continuation of culture in fresh media. De novo protein synthesis was blocked with 10 µg/mL cycloheximide alone, or in combination with the proteasome inhibitor MG-132 (5 µg/mL).

### Flow cytometry

For cell cycle analysis SB1690CB cell were pelleted after individual treatments and time points at 400 × *g* for 5 min, then washed twice with cold 1X PBS before to be resuspended in 200 µL of cold 1X PBS. Cells were fixed by drop-wise adding 800 µL of ice-cold 100% ethanol, followed by vortexing and 1-h incubation on ice. After 2 washes with FACS Buffer (1XPBS, 0.04% NaN_3_, 0.1% BSA), cells were resuspended in 0.5 mL of FACS buffer supplemented with 100 µg/mL propidium Iodine (PI) and 100 µg/mL of RNase and incubated for 30 min at 37 °C in the dark. After incubation, cells were analysed in FACS Calibur (BD) flow cytometer.

### Western blot, immunofluorescence and antibodies

Protein extracts from pelleted cells were resolved by protein electrophoresis (NuPAGE® Novex® 4–12% Bis-Tris Protein Gels, Invitrogen) and analysed by western blotting using standard methodologies.

For immunofluorescence SB1690CB cells were spun at 200 rpm for 2 min (Cytospin 2, Shandon) onto POLYSINE slides (VWR International) and fixed with methanol-free 4% formaldehyde (Thermo Scientific) for 10 min. Cells were washed in PBS and blocked with 5% goat normal serum (Cell Signaling Technology) and 0.3% Triton-X100 (Sigma) containing PBS. Primary antibodies (details listed in Supplementary Material) were used with secondary antibodies following standard procedures in a 0.1% BSA (Cell Signaling Technology) and 0.3% Triton-X100 containing PBS. Single confocal plane and sequential channel acquisitions were performed in a Fluoview1000 confocal system (Olympus), using a 60X UPLSAPO oil immersion lens. To determine levels of co-localisation of EVI1 and CtBP1, Pearson product-moment correlation coefficient (Pearson’s coefficient was used to measure the linear signal correlation (dependence) between the EVI1 and CtBP1 IF signals. Person’s coefficient ranged from 1 (total positive correlation) to − 1 (total negative correlation). 200 + circular (*r* = 1.5 mm) Regions of interest (ROI) were analysed per condition with the co-localisation plug-in of the ImageJ software. To discard signal saturated ROIs, the images were analysed using HiLo (High-Low) intensity Look Up Table (LUT). Pearson coefficients were plotted either in a dispersion graph (cell distribution in a single experiment) or as average from at least three biological replicates. One-way Analysis Of the Variance (ANOVA) with the Tukey post-test statistical analysis was used to compare the means (GraphPad Prism). Alternatively, linear ROIs of 5 mM in length were analysed in terms of signal intensity and plotted as signal histograms. Automated foci detection and counting was performed by the FociPicker3D plugin for ImageJ [7]. Briefly, nuclear ROIs were created for Individual cells at independent microscope panels and foci were detected and counted under the follow criteria: foci > 0.35 µm in diameter and a MinISetting of 0.5 (Minimum intensity setting). For the antibodies used, please refer to the figure legends and Supplementary Table 1.

### Biochemical cell fractionation

2.5 × 10^7^ SB1690CB AML cells were pelleted down at 300 × g for 4 min (4 °C) and washed 2 times in an excess of cold 1X PBS. Cell pellets were sequentially extracted with 10 volumes of a base buffer (15 mM KCl, 30 mM HEPES pH 7.4, 1 mM TCEP, 2 mM MgCl_2_, 1 mM EDTA, 1 mM Na_3_VO_4_, 1 mM PMSF, 1X Protease Inhibitor Cocktail (Sigma P8340), 1X Phosphatase Inhibitor Cocktail 2 (Sigma P5726), 1X Phosphatase Inhibitor Cocktail 3 (Sigma P0044), supplemented with: (1) 150 µg/mL digitonin (Sigma D141) and glycerol (Fisher Scientific, BP-229-1) to extract cytosol proteins; (2) 0.5% Tween-20 (Sigma P1379) and glycerol (Fisher Scientific, BP-229-1) to extract soluble organelles proteins; (3) 140 mM NaCl to extract Nucleosol proteins; 1% *n*-Dodecyl β-D-maltoside (Sigma D4641) and glycerol (Fisher Scientific, BP-229-1) to extract membrane proteins. The remnant pellet was extracted with 10 volumes of a high salt lysis buffer [420 mM NaCl, 20 mM Bicine (Sigma B3876), 2 mM MgCl_2_, 1 µM ZnCl_2_, 1 µM CaCl_2_, 0.6% CHAPS (Sigma C9426), 1 mM Na_3_VO_4_, 1 mM PMSF, 1X Protease Inhibitor Cocktail (Sigma P8340), 1X Phosphatase Inhibitor Cocktail 2 (Sigma P5726), 1X Phosphatase Inhibitor Cocktail 3 (Sigma P0044) and 250 U/µL Pierce Universal Nuclease (Thermo Scientific 88,700)] to extract chromatin associated proteins. The residual pellet was extracted with 2× LDS buffer (Invitrogen) to dissolve the nucleoskeleton. Protein extracts were resolved by protein electrophoresis using the (NuPAGE® system, Invitrogen) and analysed by western blotting.

## Results

### EVI1 is degraded during mitosis

To investigate endogenously expressed EVI1 in AML we studied 3q26 rearranged SB1690CB AML cells, which express high levels of both EVI1 and ΔEVI1, but no MDS-EVI1 (Fig. 1a) [6]. Mimosine treatment-associated G1 arrest and release resulted in reduction of both EVI1 and ΔEVI1 levels (Fig. 1b), with EVI1 only starting to recover 7 h after release (Fig. 1b). In contrast, a nocodazole-induced G2/M arrest resulted in higher EVI1 levels compared with levels at G1 arrest (Fig. 1c), which suggests that degradation of overexpressed EVI1 occurs during or shortly after mitosis (identical findings with forced EVI1 expression also in another cell line model, see Supplementary Fig. S1). To further test this hypothesis, we induced a G2/M arrest and monitored EVI1 levels over 14 h post release. We observed gradual reduction in EVI1 and ΔEVI1 levels during mitotic progression with similar patterns as CYCLIN B1 (Fig. 1c), which is degraded by the anaphase-promoting complex (APC/Cyclosome) to exit mitosis [8]. To investigate whether EVI1 degradation is also proteasome dependent, we blocked *de novo* protein synthesis with cycloheximide alone, or in combination with the proteasome inhibitor MG-132 (Fig. 1d). Cycloheximide treatment alone resulted in a marked reduction of EVI1 levels, which was partly reversed by MG-132 treatment (Fig. 1d), with patterns resembling those of CYCLIN B1 (Fig. 1e). Intriguingly, degradation of ΔEVI1 was not reversible to the same extent by MG-132, implying additional and alternative degradation dynamics for ΔEVI1.

### CtBP1 dissociates from EVI1 during mitosis

**Fig. 2:**
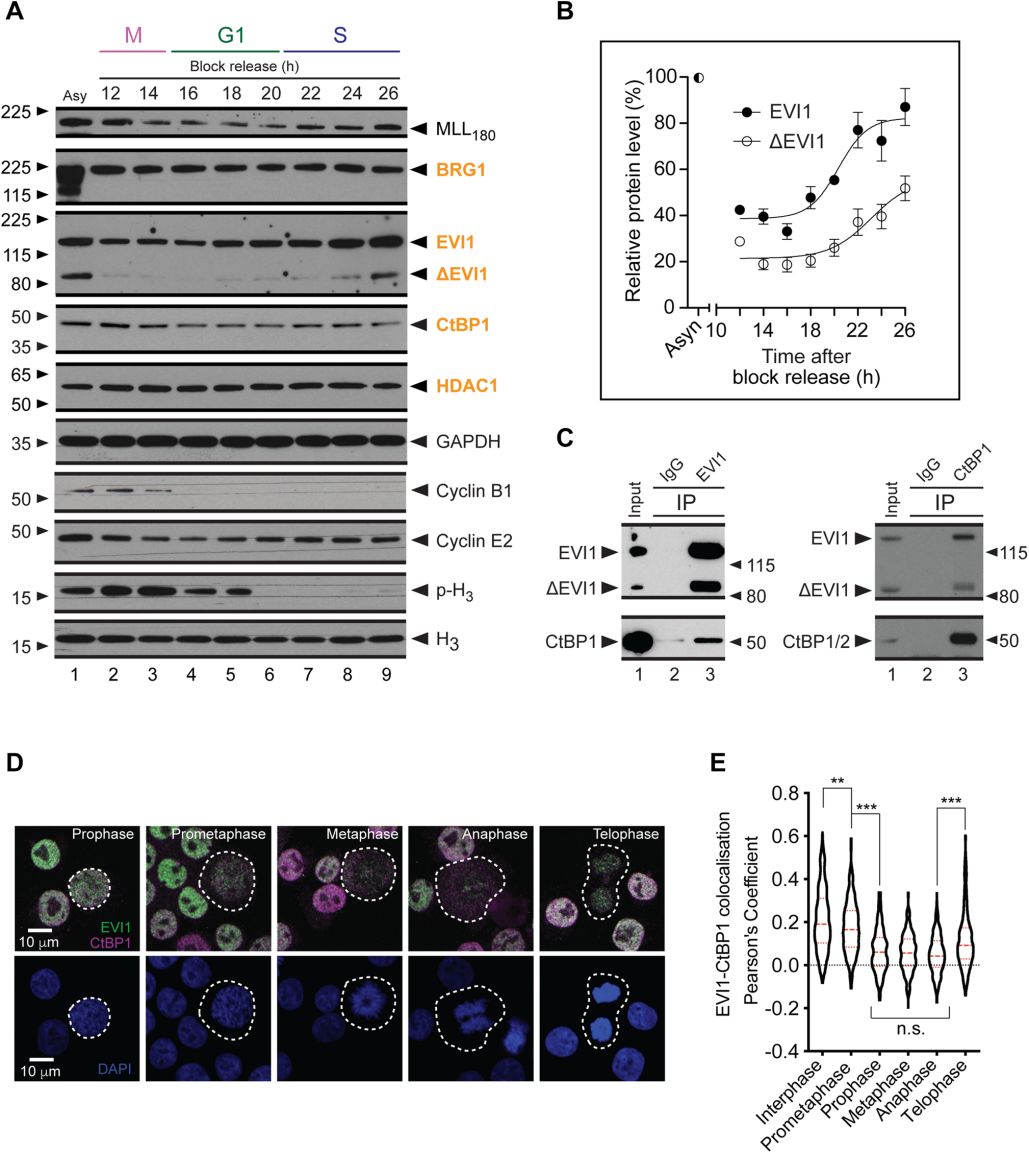
CtBP1 dissociates from EVI1 during mitosis. **a** SB1690CB AML cells were synchronised in G0/G1 with Mimosine treatment for 18 h and then released by replacement with fresh medium. Total protein extracts were produced at the time points as indicated after release from the G0/G1 block and expression levels of EVI and CtBP1 assessed by western blot. Levels of EVI1 interacting proteins BRG1 and HDAC1 were assessed as controls, Ser10-phospho H_3_ as a mitotic marker, CYCLIN B1 and CYCLIN E2 as cell cycle phase controls. MML_180_ was used as a marker which oscillates through the cell cycle and GAPDH as a loading control. **b** Quantitation of EVI1 isoform levels as in A from 3 independent experiments. **c** Co-immunoprecipitation of endogenously expressed EVI1 isoforms and CtBP1 from SB1690CB AML cells. **d** Dual colour EVI1 (green) and CtBP1 (magenta) immunofluorescence in SB1690CB AML cells. Single confocal planes acquired with a Fluoview 1000 system (Olympus). Cell cycle stage assessed by chromatin staining (DAPI, blue). Dashed lines denote cell boundaries. **e** Distribution of the Pearson Coefficient (P’sC) for the EVI1 and CtBP1 signal co-localization. 100 circular region of interests (ROI) were pooled from 5 different stains (3 µm in diameter). Statistical analysis: one-way ANOVA and Tukey post-test (n.s.=non-significant, ** *p* < 0.01, *** *p* < 0.001). (Color figure online)

G1 block and long-term release over 26 hrs confirmed oscillation of EVI1 expression during cell cycle progression, exhibiting similar patterns to the MLL protein [9], which was used as a control (Fig. 2a). Both EVI1 and ΔEVI1 levels recovered at the transition between G1 and S (calibrated by the cell cycle markers CYCLIN B1, CYCLIN E2 and p-H_3_ (Ser10) (Fig. 2b). With respect to EVI1 interacting proteins, we observed for CtBP1 similar cell cycle dependent oscillation patterns, while the EVI1 interacting proteins BRG1 and HDAC1 [10, 11] displayed stable expression levels during cell cycle progression. Both EVI1 and ΔEVI1 interact with CtBP1 (Fig. 2c), and EVI1 co-localises with CtBP1 most strongly during telophase (Fig. 2d, e).

### Differential subnuclear distribution of EVI1 and CtBP1 during interphase

**Fig. 3:**
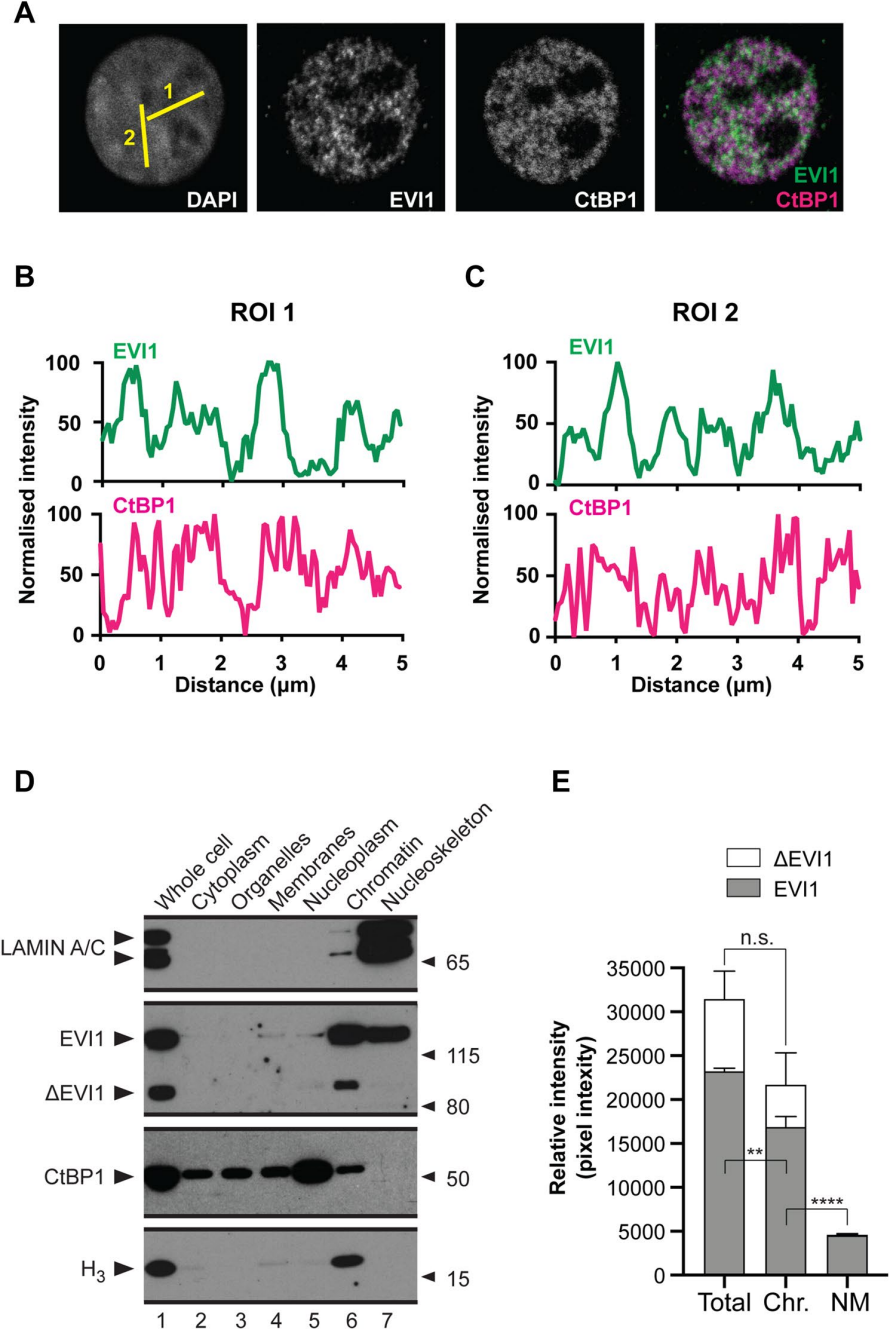
Subnuclear distribution of EVI1 and CtBP1 during interphase. **a** Dual colour EVI1 (green) and CtBP1 (magenta) immunofluorescence in AML cells. Single confocal planes acquired with a Fluoview 1000 system (Olympus) and presented individually in grey scale. Nucleus visualised by chromatin staining (DAPI). Signal intensity measured over 5 µm length (yellow lines numbered 1 and 2) linear ROIs for both, EVI1 and CtBP1 stains. **b, c** Histogram panels illustrating nuclear signal intensity distribution of EVI1 (green) and CtBP1 (purple). **d** Western blotting of AML cell fractionations and **e** Quantitation of EVI1 isoform levels in selected fraction (as in **D**) from 3 independent experiments. Statistical analysis for each isoform was performed by one-way ANOVA test and Tukey post-test (*n*.*s*. non-significant, **p < 0.01, ****p < 0.0001). (Color figure online)

We noticed different patterns of the nuclear signal distribution of EVI1 and CtBP1 in interphase: The EVI1-signal in interphase has a speckled signal distribution (Fig. 3a) in the nuclei with distinctly separable foci formations (visualised in Fig. 3b, c), whereas the CtBP1-signal was more diffuse (Fig. 3a, b, c). Foci counts (Fig. 3d) per region of interest (ROI) were significantly higher for EVI1 foci than for CtBP1 (Fig. 3e). To further determine exact protein localisation within the nucleus, which might underly these findings, on cellular fractionation we found abundant CtBP1 in the nucleoplasmic fraction (Fig. 3f) (Supplementary Fig. S2). However, EVI1, ΔEVI1, and only a fraction of CtBP1 and the EVI1-interacting proteins BRG1 and HDAC1 co-elute in the nuclear chromatin fraction (Fig. 3f, Supplementary Fig. S2). Importantly, a fraction of EVI1, but not ΔEVI1, is residing in the nucleoskeleton, evidenced by the presence of the nuclear envelop marker LAMIN A/C in that fraction (Fig. 3f, g), with similar staining patterns also during mitosis as the nucleoskeleton associated protein NuMA1 during interphase (Supplementary Fig. S3) [12].

## Discusion

High EVI1 expression in one of the most aggressive oncogenic events in leukaemia, and a similar role for aberrantly high EVI1 expression is emerging in some solid tumours [13, 14]. Understanding the functional interactions and spatiotemporal associations of the different EVI1 isoforms is therefore important. We studied endogenously expressed EVI1 and ΔEVI1 in a robust AML cell line model with a 3q26-aberration associated EVI1 overexpression [6]. Recent clinical data support the concept that all 3q-re-arranged AMLs constitute a uniform entity driven by EVI1 [2]; we therefore presume that our observations apply more generally to all EVI1-overexpressing AMLs. However, further confirmation of our findings in other cell lines and clinical samples would be important, also including EVI1-overexpressing leukaemia without 3q re-arrangements. Building on data showing that forced expression of EVI1 in haematopoietic progenitor cells inhibits normal cell cycle progression [15], here we illustrate the effect of cell cycle progression on EVI1. We can demonstrate a bimodal oscillation of EVI1 protein levels with maximum EVI1 levels at the end of S-phase, similar to that described for related transcription factors MLL and GATA2 [9, 16], and imply a role of the proteasome for EVI1-degradation, which could also provide therapeutic options for EVI1-overexpressing leukaemia. Our data further implies that the interaction with the co-repressor CtBP1, which has been shown to be essential for some EVI1 functions [5], is likely to be mainly occurring through interphase and is located in the chromatin fraction and, while a large proportion of EVI1 is located at the nuclear matrix, where we did not see CtBP1. As the repressor protein CtBP1 is considered as a therapeutic target in various cancer types [17] and might have a role specifically for EVI1 overexpressing malignancies, these observations need to be considered when targeting CtBP1 interactions therapeutically. Reported differences of functional interactions of the EVI1 isoforms with respect to transformation and protein association [4, 17] may be partly explained by their dynamic sub-nuclear localisation. Our study reports on the EVI1-CtBP1 interaction, but many more proteins have been described to interact with EVI1 [17, 18]. The detailed mechanistic understanding and the functional implications of transcription levels and protein turnover, which could be mediated by ubiquitination, sumoylation or other posttranslational modifications, and dynamic spatiotemporal interactions of EVI1 will be a critical consideration for targeted therapeutic approaches in EVI1 overexpressing leukaemia.

## Electronic supplementary material

Below is the link to the electronic supplementary material.

Supplementary material 1 (DOCX 2474 kb)

## Data Availability

All data generated or analysed during this study are included in this published article [and its supplementary information files].
