# Supplementary material for: EVI1 oncoprotein expression and CtBP1-association oscillate through the cell cycle
Source: Mol Biol Rep. 2020 Sep 26;47(10):8293–300. doi: 10.1007/s11033-020-05829-1 (PMC7588369; doi:10.1007/s11033-020-05829-1)
Supplement: Supplementary file 1 — Supplementary material 1 (DOCX 2474 kb) [file 11033_2020_5829_MOESM1_ESM.docx]

**EVI1 oncoprotein expression and CtBP1 association oscillate through the cell cycle.** Paredes *et al.*

**Supplementary Material**

**Cells:** EVI1 overexpressing acute myeloid leukaemia (AML) cells SB1690CB were established in Manchester from a 3q-rearranged AML in a 2-year-old child and maintained as described [1-3]. The interleukin-3 dependent murine pro-B cell line BAF/3 cells for additional experimental work (in Supplementary material) were initially obtained from the Gilliland/Sternberg laboratory (Boston) and continuously maintained since.

**Supplementary Table 1: Antibodies used in the study**

| **Antigen** | **Dilution range used** | **Company** | **Catalogue N^o^** |
| --- | --- | --- | --- |
| GAPDH | 1:20,000 to 1:500,000 | Ambion | AM-4300 |
| Histone H_3_ | 1:5,000 | Cell Signaling Technology | 9715 |
| pH_3_ (Ser10) | 1:5,000 | Millipore | 06-570 |
| CtBP1 | 1:2,000 | BD Biosciences | 612042 |
| EVI1 | 1:1,000 | Cell Signaling Technology | 2593 |
| Cyclin B1 | 1:1,000-1:5,000 | Millipore | 04-220 |
| Cyclin E2 | 1:1,000-1:2,000 | Millipore | 04-223 |
| HDAC1 | 1:1,000 | Cell Signaling Technology | 5356 |
| BRG-1 | 1:1,000 | Santa Cruz Biotechnology | sc-10786 |
| LAMIN A/C | 1:500 | Cell Signaling Technology | 2032 |
| SIRT-1 | 1:1,000-1:2,000 | Millipore | 05-1243 |
| RNA Pol II | 1:1,000 | Abcam | Ab-817 |
| p-RNA Pol II | 1:1,000 | Abcam | Ab-5131 |
| AML-1 | 1:200-1:1,000 | Santa Cruz Biotechnology | Sc-28679 |
| PIAS-1 | 1:1,000-1:2,000 | Cell Signaling Technology | 3550 |
| NUMA1 | 1:1,000 | Gene Tex | GTX629397 |
| MTRIN-3 | 1:200-1:1,000 | Santa Cruz Biotechnology | sc-55723 |
| CD45 | 1:500 | Abcam | ab10558 |
| RBBP4 | 1:5,000 | Abcam | ab117746 |
| GFP | 1:5,000 | Cell Signaling Technology | 2555 |
| HSP90 | 1:1,000 | Stress Gene | SP8-830 |
| FLAG | 1:1,000 | Sigma | F1804 |
| FLAG | 50 μL beads suspension /mL protein extract | Anti-FLAG^®^ M2 Magnetic Beads Sigma | M882 |
| Mouse IgG | 1:5,000-1:10,000 | Anti-mouse IgG HRP Linked Whole Ab,  GE Healthcare | NA931V |
| Rabbit IgG | 1:5,000-1:10,000 | Anti-rabbit IgG HRP Linked Whole Ab, GE Healthcare | NA9340V |
| Mouse IgG | 1:800 to 1:2,000 | Goat anti-mouse IgG (H+L), Alexa Fluor 594 conjugated, Life Technologies | A-11032 |
| Rabbit IgG | 1:800 to 1:2,000 | Goat anti-rabbit IgG (H+L), Alexa Fluor 488 conjugated, Life Technologies | A-11034 |

**Supplementary Figures**


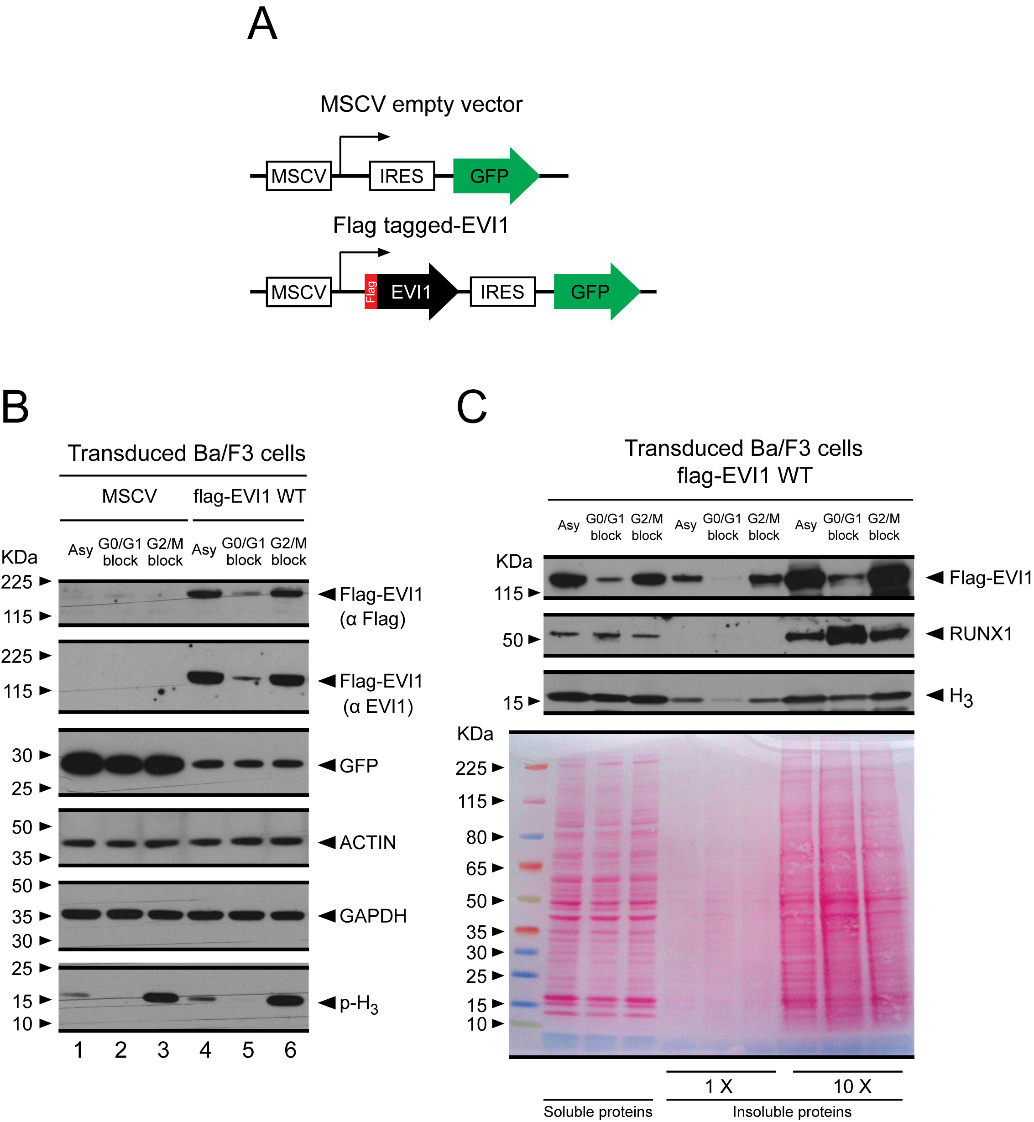


**Figure S1. Exogenously expressed EVI1 protein is cell cycle regulated. A.** Schematic of the retroviral vectors used to stable transduce murine derived Ba/F3 cells with a Flag-tagged version of EVI1 protein. **B.** Protein lysates were produced from asynchronous (Asy) cell cultures and cells arrested in G0/G1 and G2/M. EVI1 expression was detected by western blotting using anti Flag- or EVI1-antibodies. GFP was used as a transduction control, p-H_3_ as a mitosis marker and ACTIN and GAPDH as loading controls. **C.** Western blot analysis of EVI1 following a 2-step protein extraction with a high salt lysis buffer (420 mM NaCl) followed by a 2x LDS buffer extraction (insoluble proteins) of transduced Ba/F3 as in A. The insoluble fraction was run in equivalent proportion (1X) or in tenfold excess (10X) to allow protein detection. RUNX1 used control as a control that increases at G1/S and a nuclear matrix/nucleoskeleton associated protein. Ponceau S stain shown for protein loading.

**
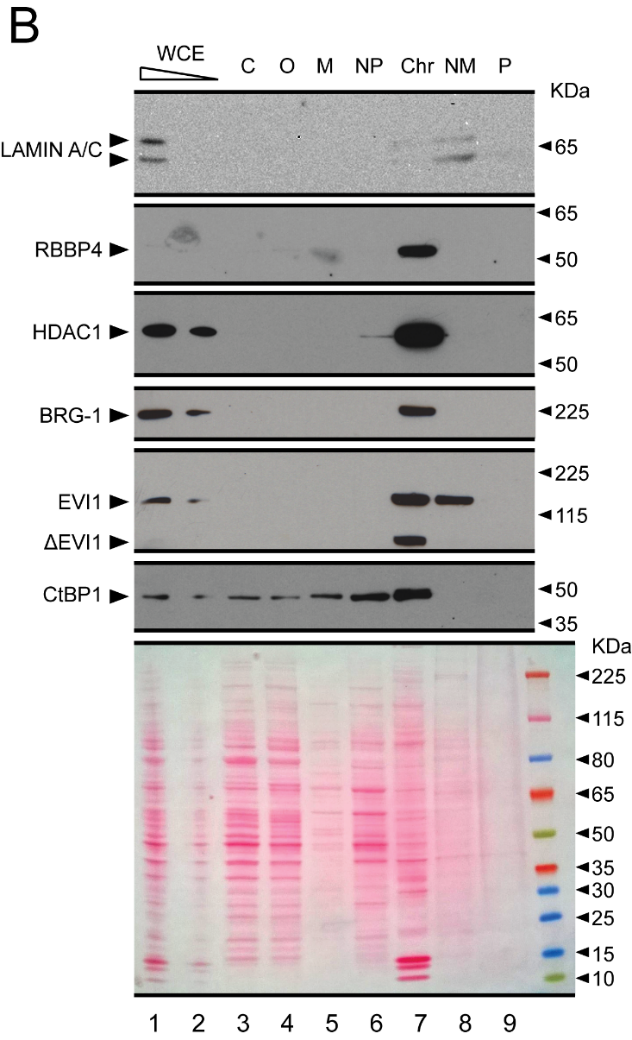

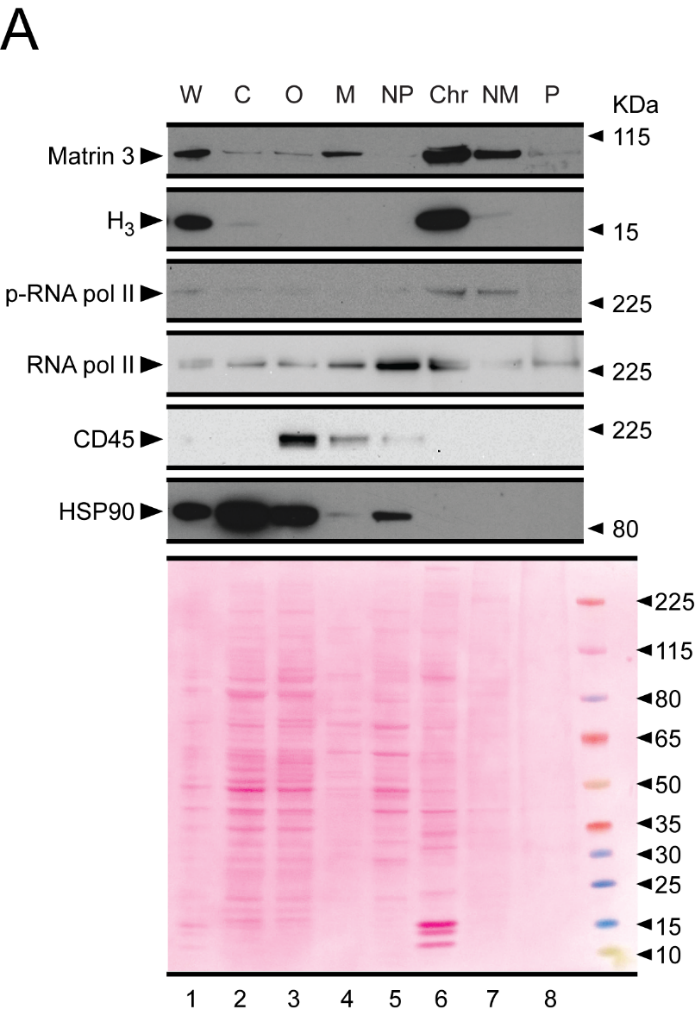
**

**Figure S2. Biochemical fractionation of SB1690CB AML cells.** **A.** Western blot analysis of the cell fractions with known subcellular markers; HSP90 (cytosol, organelles), CD45 (membranes), RNA pol II and p-RNApol II (nuclear proteins), Histone H3 (chromatin), and MATRIN 3 (chromatin and nucleoskeleton). WCE: whole cell extract, C: cytoplasm, O: organelles, M: membrane, NP: nucleoplasm, Chr: chromatin, NM: nuclear matrix (nucleoskeleton), P: remnant insoluble pellet. **B.** The cellular localisation of EVI1 and its interacting proteins CTBP1, BRG1 and HDAC1 were assessed by western blot analysis. Ponceau S stain shown for protein loading.

**
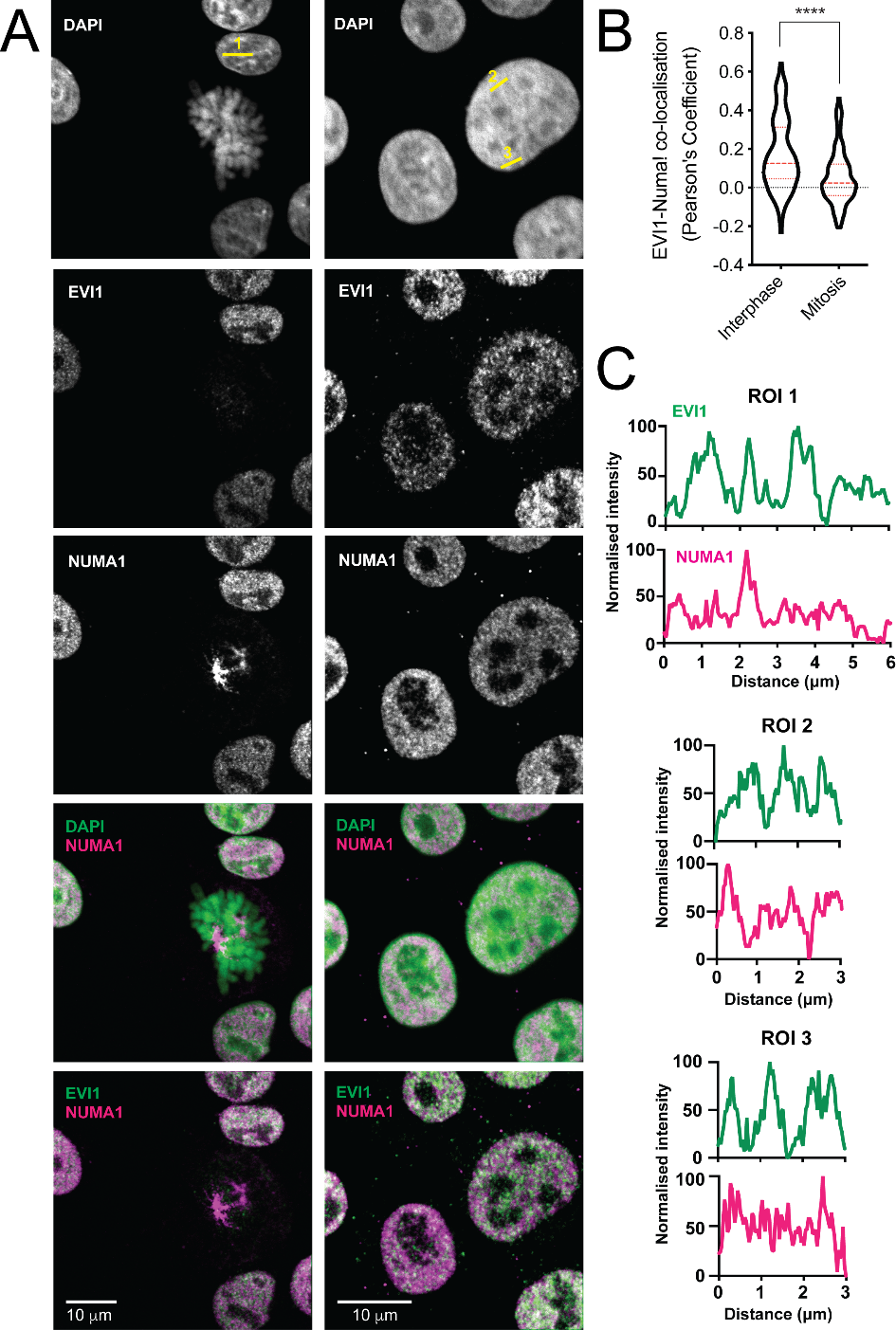
**

**Figure S3. EVI1 colocalises with NuMA1 in the interphasic nuclei.** **A.** Dual colour EVI1 (green) and NuMA1 (magenta) immunofluorescence of SB1690CB AML cells with DAPI as a nuclear co-stain with two orders of magnification shown (higher magnification in right hand panel). Signal intensities were measured in 150+ circular (*r*=1.5 µm) ROIs per condition and EVI1-NuMA1 co-localisation was determined as described previously [3] **(B).** Statistical analysis: Unpaired t test with Welch's correction (**** *p*<0.0001). **C.** Signal intensity of individual stains for EVI1 and NuMa1 in 6 or 3 µm linear ROIs (yellow lines at the top panel of each column in A).

**Supplemenatry references**

1. Meyer S, Fergusson WD, Oostra AB, Medhurst AL, Waisfisz Q, de Winter JP, Chen F, Carr TF, Clayton-Smith J, Clancy T, Green M, Barber L, Eden OB, Will AM, Joenje H, Taylor GM (2005) A cross-linker-sensitive myeloid leukemia cell line from a 2-year-old boy with severe Fanconi anemia and biallelic FANCD1/BRCA2 mutations. Genes Chromosomes Cancer 42:404-415.

2. Meyer S, Fergusson WD, Whetton AD, Moreira-Leite F, Pepper SD, Miller C, Saunders EK, White DJ, Will AM, Eden T, Ikeda H, Ullmann R, Tuerkmen S, Gerlach A, Klopocki E, Tonnies H (2007) Amplification and translocation of 3q26 with overexpression of EVI1 in Fanconi anemia-derived childhood acute myeloid leukemia with biallelic FANCD1/BRCA2 disruption. Genes Chromosomes Cancer 46:359-372.

3. Paredes R, Schneider M, Stevens A, White DJ, Williamson AJK, Muter J, Pearson S, Kelly JR, Connors K, Wiseman DH, Chadwick JA, Loffler H, Teng HY, Lovell S, Unwin R, van de Vrugt HJ, Smith H, Kustikova O, Schambach A, Somervaille TCP, Pierce A, Whetton AD, Meyer S (2018) EVI1 carboxy-terminal phosphorylation is ATM-mediated and sustains transcriptional modulation and self-renewal via enhanced CtBP1 association. Nucleic Acids Res 46:7662-7674.
